# Engineering Stable Decomposition Products on Cathode Surfaces to Enable High Voltage All‐Solid‐State Batteries

**DOI:** 10.1002/anie.202413591

**Published:** 2024-12-04

**Authors:** Lanting Qian, Yangyang Huang, Cameron Dean, Ivan Kochetkov, Baltej Singh, Linda Nazar

**Affiliations:** ^1^ Department of Chemistry and the Waterloo Institute of Nanotechnology University of Waterloo 200 University Ave Waterloo ON N2L 3G1 Canada

**Keywords:** solid-state battery, cathode-electrolyte interface, argyrodite electrolyte, DFT, cathode coating by design

## Abstract

Sulfide solid electrolytes such as Li_6_PS_5_Cl hold high promise for solid‐state batteries due to their high ionic conductivity; however, their oxidation potential of ~2.5 V is not compatible with high voltage Ni‐rich cathodes such as LiNi_x_Co_y_Mn_1−x−y_O_2_ (x≥0.8). Using guidance from density functional theory, we devise an effective, conformal, and thin coating on the cathode active material, which suppresses the oxidative decomposition of Li_6_PS_5_Cl as shown by experiment. The nanometric coating on nickel‐rich NMC85 enabled capacity retention of 82 % after 200 cycles (2.8–4.3 V vs Li^+^/Li) using Li_6_PS_5_Cl as the solid electrolyte. In comparison, cells with an uncoated CAM only displayed 56 % capacity retention. The coated‐NCM85 cells also demonstrate much better rate performance and higher capacity. The enhanced performance is due to the formation of a stable amorphous cathode‐electrolyte interphase accruing from the decomposition products of the LiPO_2_F_2_ precursor (as predicted by DFT), which protect the sulfide electrolyte from oxidation. The coating fabricated in this cost‐effective process showed superior performance to state‐of‐the‐art coatings such as LiNbO_3_. This work highlights the importance of rationally designing stable coating materials based on their potential decomposition products and confirms the suitability of a low‐cost and conformal coating to enable sulfide electrolyte‐based all‐solid‐state batteries.

## Graphical abstract

Rational design of a cost‐effective coating on high‐voltage NCM85 cathode for all‐solid‐state batteries—utilizing the stable decomposition products of LiPO_2_F_2_—successfully suppresses the electrochemical decomposition of the argyrodite Li_6_PS_5_Cl solid electrolyte. Cells using the LiPOF‐coated NCM85 demonstrated significantly improved capacity retention over 200 cycles, outperforming cells using uncoated NCM85.

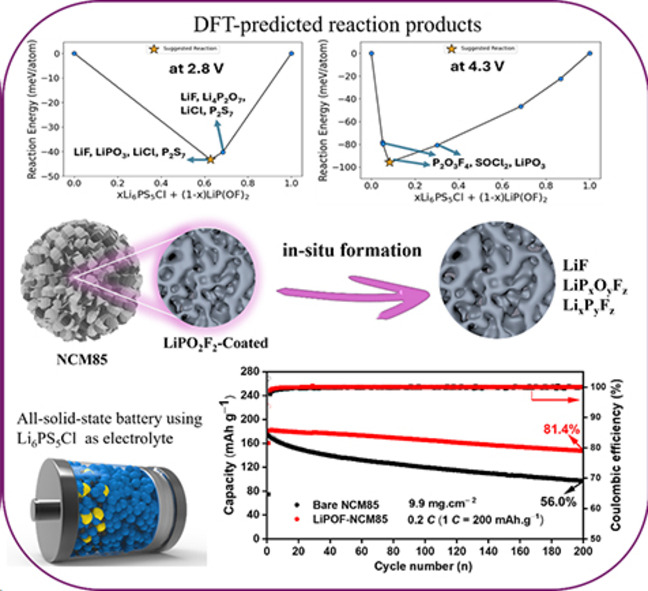

## Introduction

Solid‐state batteries (SSBs) have garnered significant interest in the realm of next‐generation energy storage devices because of their potential to achieve greater energy density and superior safety performance in comparison to state‐of‐the‐art lithium‐ion batteries (LIBs).[^1^, ^2^] The development of high‐performance solid‐state cells is contingent upon several factors, including the utilization of solid electrolytes (SEs) that have favorable interfaces with the electrodes and high ionic conductivities at room temperature.[^3^, ^4^, ^5^] Sulfide electrolytes such as argyrodite (Li_6_PS_5_Cl) are electrochemically quasi‐stable against reduction by a Li anode due to the formation of an interphase consisting of Li_2_S, Li_3_P, and LiX (where X=Cl, Br).[^6^, ^7^, ^8^] This interphase functions as a protective passivating layer, although dendrite growth is still a challenge when using sulfide electrolytes unless the solid is >95 % dense.^[9]^ In contrast, using Li−In as an anodemitigates dendrite growth and reduces chemical reactivity, which makes it useful for studying the cathode side of the battery without the effects of anode instability.

At the positive electrode, however, sulfide catholytes are oxidized at a potential of ~2.5 V vs Li^+^/Li, which leads to rapid capacity loss when used in conjunction with high‐voltage cathodes operating up to 4.3 V.[^10^, ^11^] This capacity fading is caused by a build‐up of interfacial decomposition products (PO_x_ and SO_x_) between the solid electrolyte and the cathode active material (CAM) that increases the cell resistance. However, the electrochemical stability of any SE with the CAM can be extended kinetically in the presence of stabilized interfacial products. For instance, LiPON is compatible with high‐voltage cathodes due to the high oxidative stability of its decomposition products (Li_4_P_2_O_7_, LiPO_3_, P_4_O_10_).^[7]^ Such a concept can be extended to designing coatings so that their decomposed products are stable and passivate the cathode surfaces. In principle, an ideal coating should possess not only a suitable electrochemical window (either the coating material's intrinsic electrochemical window or the electrochemical window of its kinetically stabilized products) but also meet the criteria of low electronic conductivity, high ionic conductivity, and the ability to form a thin and homogeneous layer on the cathode.

Motivated by this concept, we utilize density functional theory (DFT) to examine lithium difluorophosphate—LiPO_2_F_2_ (LiPOF), which has been used as a soluble additive in liquid cells to aid in the formation of a stable cathode‐electrolyte interphase (CEI).[^12^, ^13^, ^14^] The use of additives and polymer materials to form a stable CEI has mostly been reported in liquid cells[^15^, ^16^] but has seen application in solid‐state cells in a few recent studies.[^17^, ^18^, ^19^, ^20^, ^21^] Our computational results and experimental validation show that the formation of favourable LiF and LiP_x_O_y_F_z_ interfacial products can effectively suppress Li_6_PS_5_Cl decomposition. We utilize a simple solution process to coat LiPOF onto the CAM, NCM85, to form a nanometric coating layer. The process is carried out under ambient atmosphere without high‐temperature sintering, which is beneficial for scalability. In comparison, conventional coatings such as LiNbO_3_, Li_2_ZrO_3_, and Li_3_BO_3_‐Li_2_CO_3_ are generally applied by spray‐coating or sol‐gel methodsthat involve sintering (>300 °C), making them more costly.^[22]^ Our process takes advantage of the solubility of LiPO_2_F_2_ salt in common polar solvents that are easily evaporated. The decomposed products (typically insoluble in common solvents) deposit onto the NCM surface. A cell containing LiPOF‐coated NCM85 at an areal capacity of 1.8 mAh.cm^−2^ and Li_6_PS_5_Cl as the SE retains 81 % of its capacity after 200 cycles at room temperature, compared to only 56 % capacity retained by the cell with uncoated CAM. Furthermore, a high‐loading cell with LiPOF coating exhibits a reversible capacity of ~4.4 mAh.cm^−2^ and retains 77% of its capacity over 200 cycles. The LiPOF‐coated NCM85 cell provides electrochemical performance that is comparable to—if not superior to—that of the current inorganic and polymer coating materials.[^22^, ^23^, ^24^, ^25^, ^26^, ^27^, ^28^, ^29^] This showcases the potential of this conformal, simple, and cost‐effective coating in advancing the capabilities of all‐solid‐state batteries (ASSBs).

## Results and Discussion

**Figure 1:**
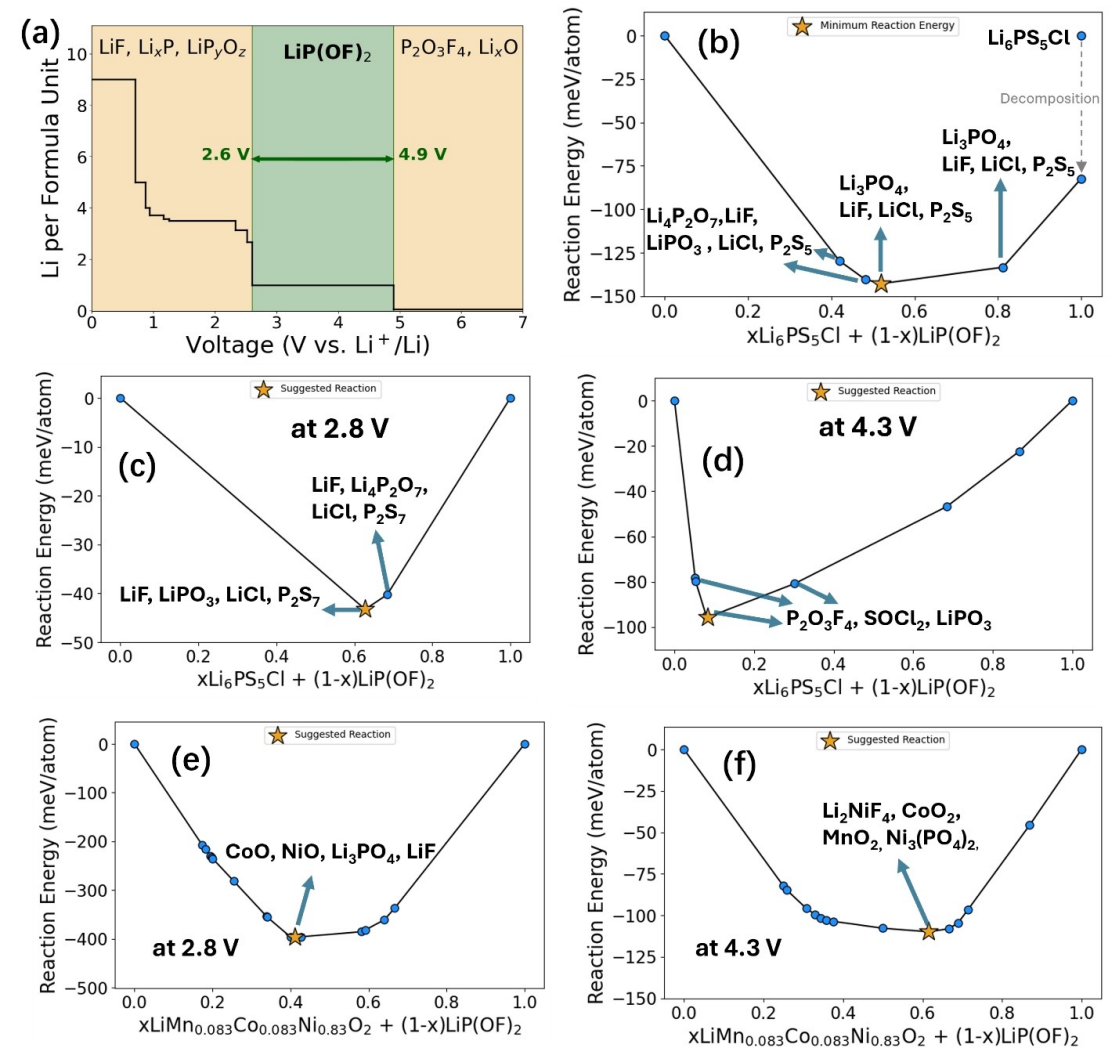
Theoretical investigation of LiPOF as a solid‐state coating using DFT calculations. a) Electrochemical stability range of LiPOF. b) Thermodynamic evaluation of interfacial reactivity between Li_6_PS_5_Cl and LiPOF without applied potential and at c) 2.8 V and d) 4.3 V vs Li^+^/Li. e) Thermodynamic evaluation of interfacial reactivity between NCM and LiPOF at e) 2.8 and f) 4.3 V vs Li^+^/Li.

LiPOF was chosen because it has been reported to inhibit alkylcarbonate oxidation when used as an additive in liquid‐electrolyte LIBs).[^9^, ^10^, ^11^] Inspired by that concept, we utilized DFT to assess the viability of LiPOF to be applied in a solid‐state system by evaluating its electrochemical stability and chemical compatibility with the Li_6_PS_5_Cl SE. First, we compared the bulk energies of materials within the Li‐P‐O‐F chemical system to construct grand potential phase diagrams. We evaluated the thermodynamic stability of LiPOF across a wide range of Li chemical potential to identify the voltage limits within which the grand potential of LiPOF lies on the convex energy hull. Following this method, Figure 1a shows that LiPOF is thermodynamically stable under applied potentials from 2.6 to 4.9 V. This voltage range encompasses the typical potentials used for NCM85 cathodes (2.8–4.3 V vs Li^+^/Li), indicating that the LiPOF coating is intrinsically stable under normal operating conditions.

While the voltage stability window of this material provides valuable information regarding its performance as a bulk material which is isolated from contact with other chemical species, it fails to account for reactions that may occur between this material and the electrolyte or cathode. These reactions can consume arbitrary amounts of either phase; therefore, we consider the possible reactions in the form of xP1+(1‐x) P2→P3, where x denotes the mixing ratio of either phase; P1, and P2 denote the two phases in contact; and P3 represents the interphase that is formed. Figure 1b shows the low energy phase that can form between Li_6_PS_5_Cl and LiPOF in the form of xLi_6_PS_5_Cl+(1‐x)LiPOF → P3. When *x*=0.52, the reaction is most energetically favourable, forming Li_3_PO_4_, LiF, LiCl, and P_2_S_5_. At slightly different mixing ratios, (0.4<*x*<0.6), several other favourable products such as LiF, LiPO_3_, and Li_4_P_2_O_7_ are predicted. The energy at *x*=1.0 is plotted in Figure 1b for the combination of Li_3_PS_4_, Li_2_S, LiCl, which is predicted to be thermodynamically more stable than Li_6_PS_5_Cl, consistent with the findings of Wagemaker et al.^[30]^ and Jain et al.^[31]^ **Table S1** shows the electrochemical window of the products formed which are extracted from other works[^7^, ^32^, ^33^], or estimated using DFT. These materials have shown excellent properties as coating materials[^6^, ^34^] and therefore are promising species to create at the electrolyte interface. These calculations were extended by treating lithium as an open element. As an open element, the lithium chemical potential is held constant, and the reaction energy is calculated using the same method as above under this restriction. Figure 1c,d displays the reactions of the formed products at 2.8 V and 4.3 V. At 2.8 V, the most favorable reactions yield LiPO_3_, LiF, LiCl, and P_2_S_7_ as products. All have much lower reactivity than Li_6_PS_5_Cl against NCM. At 4.3 V (Figure 1d), the favorable decomposition products are LiPO_3_, P_2_O_3_F_4_, and SOCl_2_. We expect that lithiation/reduction of the P_2_O_3_F_4_ will occur on discharge at 2.8 V to form LiP_2_O_3_F_4_ or “amorphous LiP_x_O_y_F_z_” (as indeed confirmed by XPS; see below).

In particular, the combination of LiF (formed at 2.8 V), LiPO_3,_ and LiP_x_O_y_F_z_ are expected to generate a stable interphase at the electrolyte/coating surface based on previous studies.[^18^, ^35^] The decomposition to liquid SOCl_2_ is a kinetically limited reaction, which is unlikely to occur. Given that these products are chemically stable and exhibit high voltage stability windows (see **Table S1**), they are expected to passivate the surface by preventing further reaction between the coating and electrolyte. In both cases, the reaction energies are predicted to be less than 100 meV/atom, which has been used as an indicator for low chemical reactivity.^[7]^ Thus, we predict that LiPOF has a wide voltage stability window and good compatibility with the solid‐state Li_6_PS_5_Cl electrolyte.

Figure 1e,f displays the reactions of the products formed between NCM and LiPOF at 2.8 V and 4.3 V. At 2.8 V, the most favourable reactions yield CoO, NiO, Li_3_PO_4_, LiF; and at 4.3 V, CoO_2_, MnO_2_, Li_2_NiF_4_, and Ni_3_(PO_4_)_2_ are predicted as products. As we don't detect many of these products on the cathode in any measurable quantity, we expect that despite the large reaction energies, the reactions are self‐limiting and the extent of decomposition is restricted by kinetics—as suggested by our experimental results (see XPS and ToF‐SIMs, below). In addition, it is expected that the interfacial products formed between the CAM and the LiPOF will have little impact on the degradation of Li_6_PS_5_Cl as the argyrodite crystallites will be primarily in contact with the interfacial products formed between Li_6_PS_5_Cl and LiPOF. All in all, these calculations predict the thermodynamically favourable decomposition products but neglect the impact of reaction kinetics, which is critically important for interfaces where the reactive species are significantly constrained. As such, we use these results as low‐cost predictions of compounds that may form at the interface of the LiPOF coating and electrode/electrolyte interface, and perform experimental measurements for a thorough analysis.

**Figure 2:**
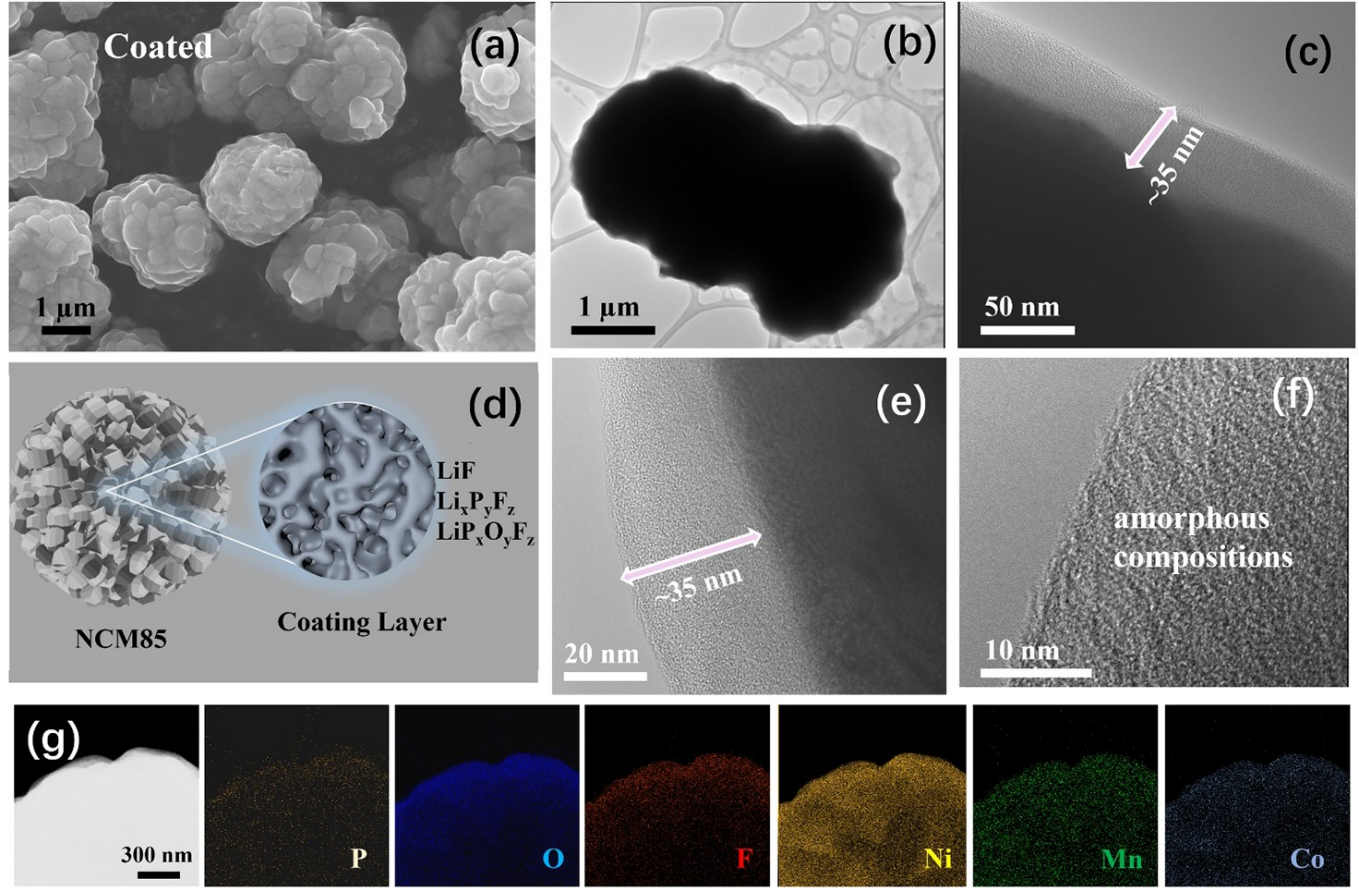
Visualization of coating on NCM. a) SEM image of LiPOF‐coated NCM particles. HRTEM images of LiPOF‐coated NCM85 at b), c) low magnification and e), f) high magnification. g) EDX mapping of the coated NCM85.

LiPO_2_F_2_ was applied to the surface of NCM85 via a straightforward wet coating method (see Supporting Information). During the coating process, the LiPOF salt partially decomposes into stable products on the NCM surface. Fourier‐transformed infrared spectroscopy (FTIR) spectra (**Figure S1**) show that the characteristic bands of LiPOF are visible in LiPOF‐coated NCM but absent in bare NCM. The bands at 1273 and 1163 cm^−1^ are assigned to the asymmetric and symmetric stretches of the P−O bonds, respectively.^[35]^ The asymmetric and symmetric stretches of the P−F bonds are reflected in the bands at 934 and 887 cm^−1^, respectively.^[35]^ X‐ray photoelectron spectroscopy (XPS) was employed as a more sensitive and selective technique in an attempt to observe the partial decomposition of LiPOF to LiF and Li_x_P_y_F_z_ via the F 1s XPS (**Figure S2**) spectrum. However, these results show that the material before and after coating is mostly LiPOF and LiP_x_O_y_F_z_. X‐ray absorption near edge structure (XANES) and powder X‐ray diffraction (PXRD) confirm that NCM85′s bulk hexagonal layered R3^−^m structure remains unchanged before and after coating, as expected (**Figure S3, S4**). Scanning electron microscopy (SEM) images also show that both uncoated and coated NCM85 have a similar spherical structure (Figure 2a and **S5**) with a diameter of ~4 μm composed of primary crystallites that make up the secondary particles. High‐resolution transmission electron microscopy (HRTEM) images (Figure 2b,c,e,f) of LiPOF‐coated NMC85 show a uniform amorphous coating that is ~25–35 nm thick on the surface of the primary NMC85 crystallites. Energy dispersive X‐ray spectroscopy (EDX) mapping (Figure 2g) of the LiPOF‐coated NCM85 shows that coating materials comprised of Li, P, O, and F are uniformly distributed throughout the surfaces of the CAM. Additional HRTEM images and EDX maps of the coating on the NCM particles are displayed in **Figure S6**.

**Figure 3:**
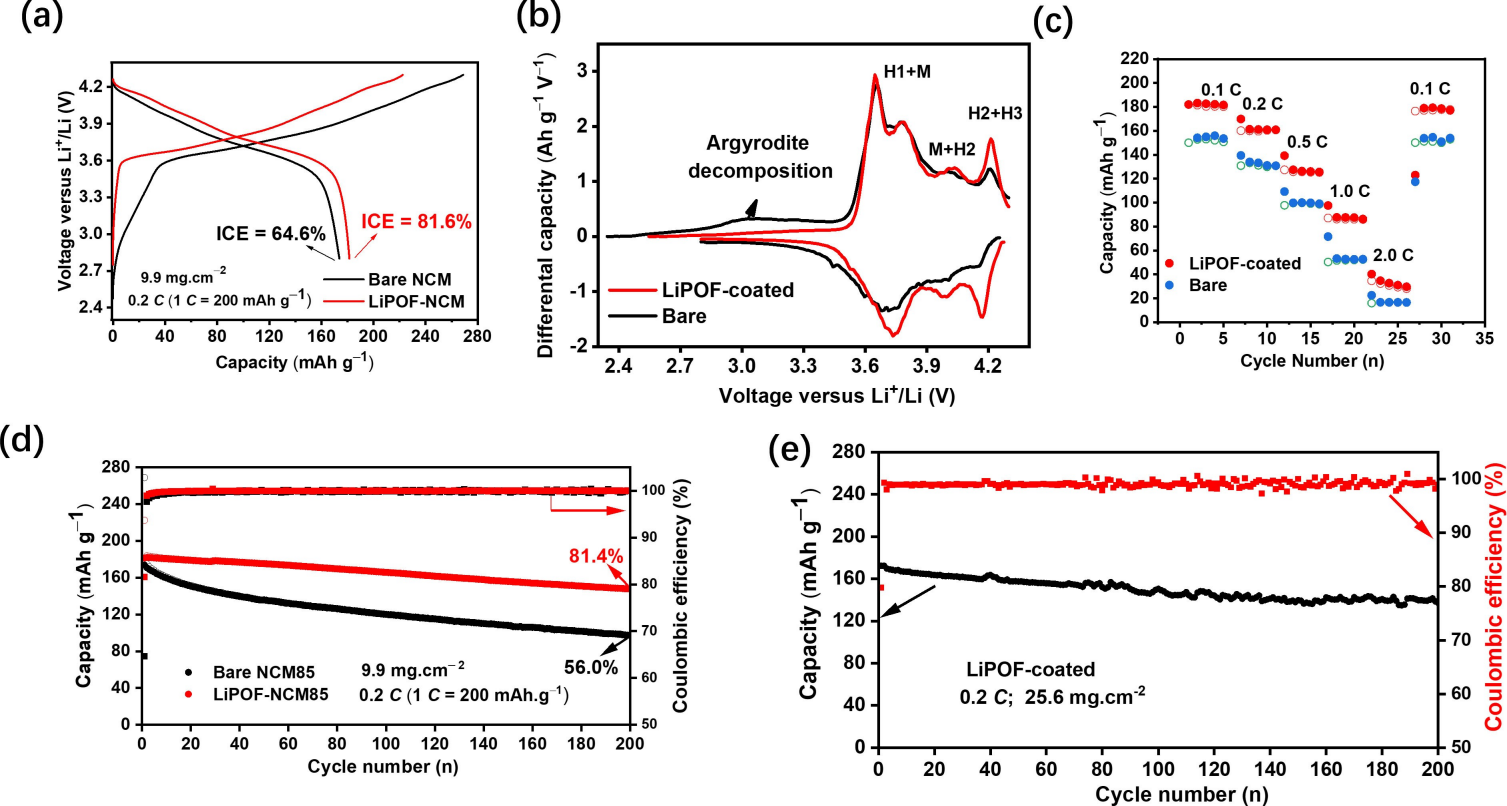
Electrochemical performance of the bare and LiPOF‐coated SSB. a) Rate performance of bare NCM85 and LiPOF‐coated NCM85 cells. b) Initial charge/discharge profiles of bare NCM85 and LiPOF‐coated NCM85 at 0.2 *C* within the voltage range of 2.8–4.3 V vs Li^+^/Li. c) Corresponding dQ/dV curves. d) Comparison of cycling performance of bare NCM85 and LiPOF‐coated NCM85 cells at moderate loading. e) Cycling performance of LiPOF‐coated NCM cell at high loading (25.6 mg.cm^−2^).

ASSBs were assembled using LiPOF‐coated or bare NCM85 as the cathode, commercial Li_6_PS_5_Cl as the SE, and In/LiIn as the anode. The (commercial) Li_6_PS_5_Cl SE has an ionic conductivity of 2.2 mS.cm^−1^ and particle size of ~1 μm. The first charge/discharge cycle was used to evaluate the initial coulombic efficiency (CE) and the effectiveness of the coating material. Figure 3a shows the initial charge and discharge profiles of pristine and coated NCM85 at a rate of 0.2 C in the voltage window 2.8–4.3 V vs Li^+^/Li. The coated cathode delivers improved discharge capacity (182 vs 170 mAh.g^−1^) and a much higher CE (82 % vs 65 %), suggesting inhibited interfacial reactivity between NCM85 and Li_6_PS_5_Cl. The profile for uncoated NCM85 exhibits a distinctive sloping region below 3.5 V on initial charge (Figure 3b), which corresponds to the oxidation of Li_6_PS_5_Cl.^[36]^ That sloping region is absent in the voltage profile of LiPOF‐coated NCM85, proving that the coating inhibits the oxidation of Li_6_PS_5_Cl. The result is highlighted by the associated d*Q*/d*V* profile of LiPOF‐coated NCM85 (Figure 3b). The initial profile observed for the uncoated NMC85 cell indicates argyrodite oxidation/decomposition that is not present in the d*Q*/d*V* plot of the coated cell. During the delithiation of NCM85, three redox peaks correspond to the well‐known H1 to M, H2, and H3 phase transitions.[^37^, ^38^] In contrast to uncoated NCM85, LiPOF‐coated NCM85 exhibits higher and more reversible peak intensities, especially for the H2+H3 transition. While we don't fully understand this finding, it indicates that the stable electrode‐electrolyte interphase resulting from the incorporation of LiPOF and its decomposition products favors reversible electrochemical processes.

Figure 3c displays the rate capabilities of the coated and uncoated NCM85 in ASSBs. The 1.0 wt % LiPOF coated‐NCM85 delivers high discharge capacities of 180 mAh.g^−1^ and 85 mAh.g^−1^ at 0.1 *C* and 1 *C*, respectively. In comparison, the uncoated NCM85 only delivers 152 mAh.g^−1^ and 51 mAh.g^−1^ at the same rates. Electrochemical oxidation of Li_6_PS_5_Cl during cycling induces the formation of insulating PO_x_ and SO_x_ phases on NCM85, thus increasing the internal resistance of the cell. As a consequence, the uncoated NCM85 cell demonstrates the least efficient rate performance. The long‐term cycling performance of coated and uncoated NCM85 cells at moderate loading (~1.8 mAh.cm^−2^) is displayed in Figure 3d. The coated NCM85 (1 wt % LiPOF) maintains a high capacity of 147 mAh.g^−1^ after 200 cycles at 0.2 C, which corresponds to 81 % retention (90 % retention after 100 cycles). On the other hand, the uncoated NCM cell delivers only 97 mAh.g^−1^ capacity with 56 % retention after 200 cycles. We note that higher LiPOF content was not beneficial: 2 wt % LiPOF‐coated NCM85 (**Figure S7**) offers stable cycling performance similar to 1 wt % LiPOF coated NCM (92 % vs. 93 % retention after 80 cycles), but lower initial capacity (~167 *vs* 182 mAh.g^−1^) due to increased cell impedance from the thicker layer. The performance of the LiPOF‐coated SSB is comparable or even superior to SSBs using well‐developed coatings^[22]^ such as LiNbO_3_,[^39^, ^40^] Li_2_ZrO_3_,^[41]^ Li_3_BO_3_‐Li_2_CO_3_,^[25]^ and HfO_2_,^[42]^ making it very promising given the low cost and ease of deposition. For instance, in studies (also using Li_6_PS_5_Cl as the SE; NCM85 as the cathode and a comparable cathode loading with a 0.2 *C* rate), LiNbO_3_‐coated cathode ASSBs displayed a capacity retention of 84 % after 75 cycles,^[40]^ while Li_2_HfO_3_/HfO_2_ coatings provided retention of 81 % after 200 cycles (but at 45 °C).^[42]^ Commercially‐coated NCM materials may differ. Relative to recent reports that use protective additives or polymer materials in SSBs, our approach is also quite promising. We achieve superior capacity (182 mAh.g^−1^ to 148 mAh.g^−1^) and capacity retention (90 % vs 80 % after 100 cycles at the same *C*‐rate) compared to lithium difluorobis(oxalato)phosphate)‐coated NCM622.^[18]^ Shi et al. showed stable cycling with the polymer coating poly ((4‐vinyl benzyl)trimethylammonium bis(trifluoromethanesulfonylimide)) at a similar cathode loading as our work, retaining 86 % capacity after 100 cycles at a slower rate of 0.1 *C*.^[17]^ Very recently, Kim et al. dry‐mixed LiPO_2_F_2_ as an additive with NCM811 to produce a bimodal powder that reduced interparticle voids in the sheets and subsequent particle cracking in dry electrodes, with promising results in high‐loading cells at 60 °C (although capacity retention was not improved relative to cells without the additive).^[19]^ Although our cells performed well at a loading of ~10 mg.cm^−2^, areal capacities of >3 mAh.cm^−2^ are required to be competitive with commercial LIBs. Indeed, a high‐loading cell (25.6 mg.cm^2^) exhibited an areal capacity of 4.4 mAh.cm^−2^, which showed an initial discharge capacity of 173 mAh.g^−1^, and retained 77 % capacity after 200 cycles at 0.2 *C* (Figure 3e).

**Figure 4:**
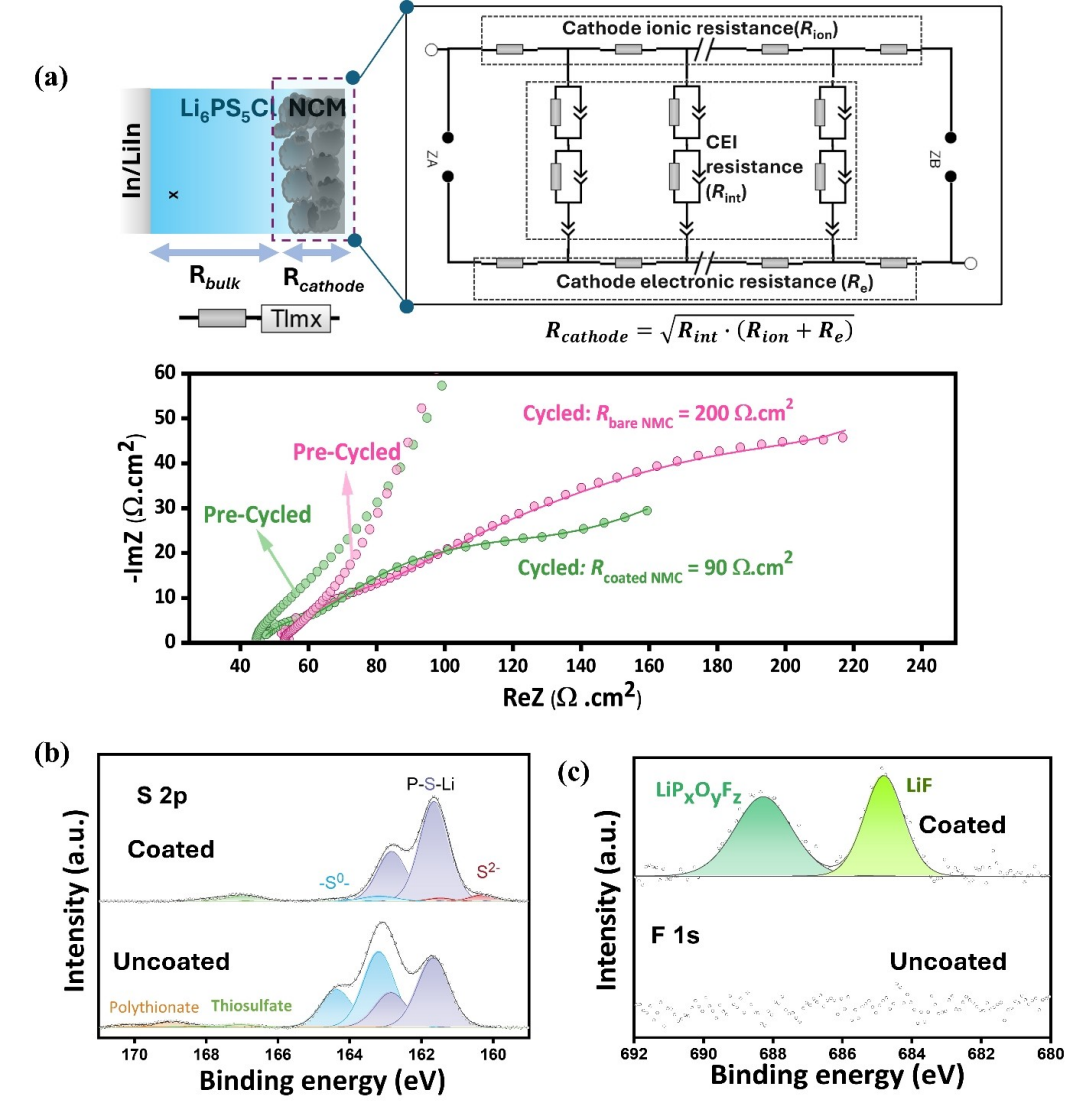
Origin of enhanced performance of LiPOF‐coated SSB. a) Nyquist plots of the bare and LiPOF‐coated NCM85 before and after cycling. Cycled cells are fitted with a transmission line model. b) S *2p* XPS spectra and c) F 1s spectra of the bare and LiPOF‐coated NCM85 cells after 200 cycles. EIS was recorded each cycle at OCV (~3.7 V vs Li−In) after discharging the cycled cells at 2.8 V.

To better understand the decline in cell performance resulting from the degradation of the SE and CAM, electrochemical impedance spectroscopy (EIS) was performed on the coated and uncoated cells before and after 200 cycles at 0.2 *C*. Prior to cycling, the cells show similar bulk resistance compared to that of the cycled cells. The semicircle features in the Nyquist plots attributable to cathode resistance are small (estimated:<10 Ω.cm^2^) and overlap with the diffusion regime of the Li^+^, and hence cannot be fitted accurately. Nevertheless, comparison of the pre‐cycled and cycled data indicate that the change in resistance for the coated cell is much less. The Nyquist plots of the cycled cells exhibit three frequency‐separated features: a high‐frequency onset (>105 Hz), a depressed semicircle between 105 and 0.5 Hz, and a short tail at a frequency below 0.5 Hz. The equivalent circuit employed to fit the EIS data is shown in Figure 4a. It consists of a bulk solid electrolyte resistance (R_bulk_) and a cathode resistance (R_cathode_). R_cathode_ values for the coated and uncoated cathodes were determined using a transmission line model with blocking boundary conditions that was used to fit the experimental data (Figure 4a), following the procedure described in our previous study.^[43]^ Upon cycling for 200 cycles at 0.2 C, the growth of the decomposed product results in larger resistance. In good agreement with the better capacity retention of the LiPOF‐coated NCM85 cell, its R_cathode_ (90 Ω.cm^2^) is less than one‐half of the uncoated NCM85 cell (200 Ω.cm^2^). The significant difference indicates that the coating effectively suppresses the oxidative decomposition of Li_6_PS_5_Cl, which is also proven by ex situ XPS and time‐of‐flight secondary ion mass spectrometry (ToF‐SIMS), as discussed below.

Figure 4b shows the S *2p* XPS spectra of the uncoated and coated NCM85 cells after 200 cycles. The S *2p* spectrum of the coated NCM85 exhibits three distinct doublets defined according to their 2p_3/2_ binding energies. The signal observed at 160.2 eV is attributed to the “free” S^2−^ ions in Li_6_PS_5_Cl,[^10^, ^44^] and the signal at ~161.6 eV is assigned to the PS_4_^3−^ tetrahedra of argyrodite.^[7]^ The tiny peak at ~163.2 eV corresponds to sulfur formed via oxidation of argyrodite.^[7]^ This negligible peak in the coated NCM85 spectra—and very significant contribution in the uncoated NCM85—confirms that LiPOF effectively suppresses the oxidation of Li_6_PS_5_Cl. The result is consistent with the lower R_cathode_ of the LiPOF‐coated NCM85 cell. The F 1s XPS spectra (Figure 4c) were analyzed to probe the change in the composition of the coating after 200 cycles at 0.2 *C*, referencing the LiP_x_O_y_F_z_ and LiF peaks to the literature.[^19^, ^35^] In comparison to the pre‐cycled cell (**Fig.S2**), it is clear that much of the LiPOF and Li_x_P_y_F_z_ were converted to LiF and LiP_x_O_y_F_z_ (Figure 4c). This corroborates the computational results. P *2p* XPS spectra of pristine LiPOF‐coated NCM particles and cycled composite cathodes (coated and uncoated) after 200 cycles at 0.2 *C* are displayed in **Figure S8**. While computation suggests the formation of LiPO_3_ from decomposed LiP_x_O_y_F_z_, P *2p* XPS spectra cannot definitively identify its presence since its binding energy is the same as PO_x_ species that may accrue from partial oxidization of Li_6_PS_5_Cl. However, the absence of significant sulfide oxidation products in the S *2p* spectrum (Figure 4b) indicates that the argyrodite is *not* oxidized, and hence the P‐O_x_ species at 133 eV in the P *2p* XPS spectrum of the cycled coated cell (**Figure S8**) is likely LiPO_3_.

**Figure 5:**
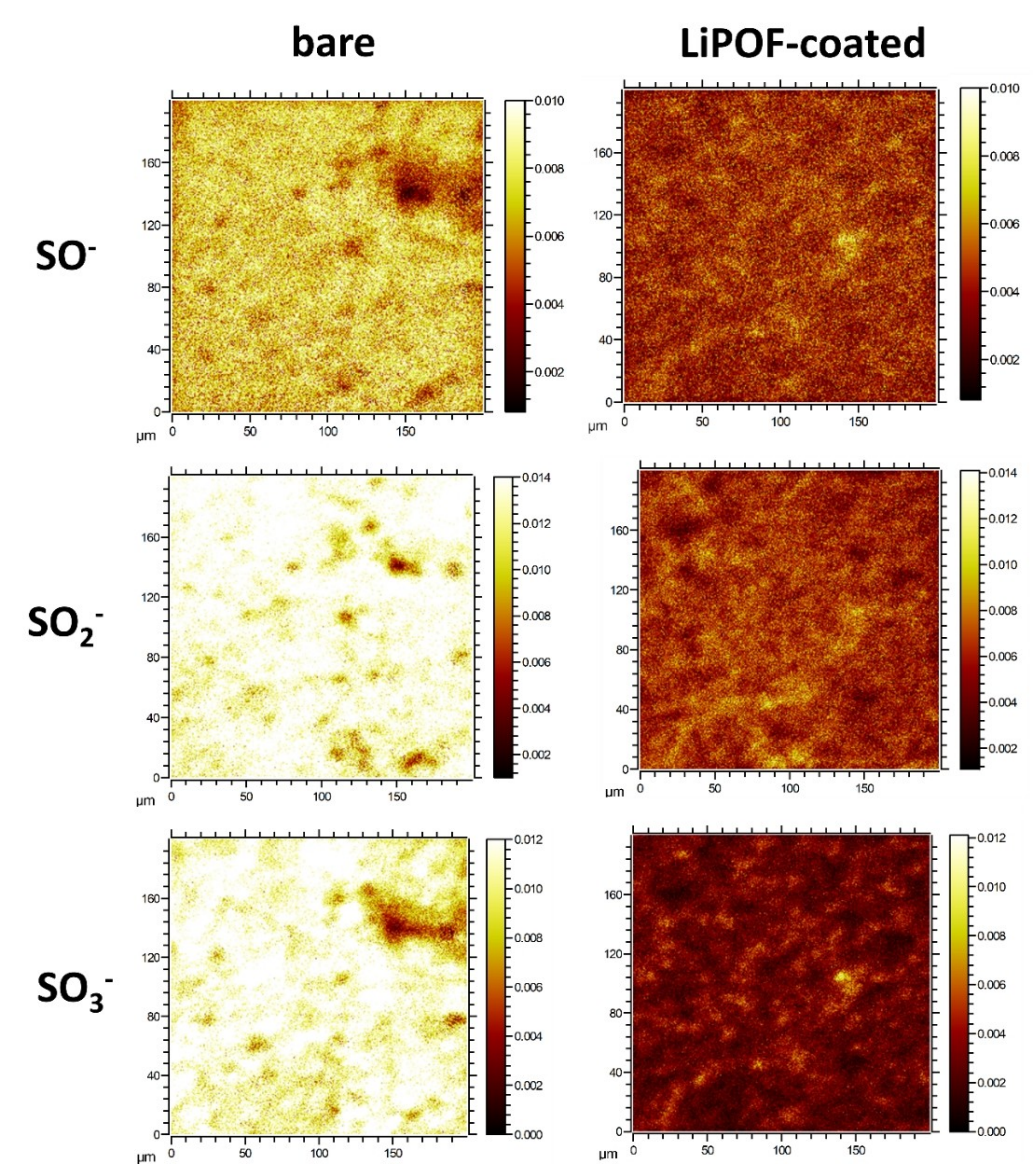
Comparison of ToF‐SIMS secondary images of negatively charged SO_x_^−^ fragments of bare and LiPOF‐coated NCM85 composite cathodes. As the ToF‐SIMS images display a large sample area of 200×200 μm^2^ which contains numerous NCM85 particles (D50=5 μm), numerous SE‐NCM85 interfaces are included to give unbiased results.

ToF‐SIMs was used as a complementary tool to XPS due to its higher sensitivity by several orders of magnitude, and its ability to identify and semi‐quantify molecular fragments.^[45]^ The relatively large analysis area minimizes bias due to the measurement of a small number of cathode and electrolyte particles. We analyzed the SO_x_^−^ (1≤x≤3) fragments which are ascribed to the products formed by the chemical reaction between delithiated NCM and Li_6_PS_5_Cl. The relative intensity of the secondary‐ion signals was normalized to the total ion signals, and all signal intensities were collected from the corresponding normalized secondary‐ion images. The ToF‐SIMS images display a large sample area of 200×200 μm^2,^ which contains numerous NCM85 particles (D50=5 μm), hence encompassing a multitude of SE‐NCM85 interfaces. Figure 5 compares the heat maps of all SO_x_^−^ (1≤x≤3) species for the uncoated and coated composite cathodes. As evident in the SO_x_^−^ maps, the average intensity of the oxidized sulfide species of the uncoated‐NCM85 and Li_6_PS_5_Cl composite is *much* higher than that of LiPOF‐coated NCM composite, which indicates that the degradation is much more pronounced in the uncoated NCM85 cell.

The SO_x_^−^ signals indicate different oxygen‐involving‐degradation processes that are dominated by the diffusion‐controlled reaction between NCM and SE, as previously documented.[^46^, ^47^] For the uncoated composite cathode, the normalized intensity of the fragments ranks in the order of SO_2_^−^>SO_3_^−^>SO^−^, whereas it is SO_2_^−^>SO^−^>SO_3_^−^ for the coated cathode. The relative ratio for individual fragments of uncoated to coated ranks in the order: SO_3_^−^>SO_2_^−^>SO^−^, which means the coating is very effective in preventing sulfur from being oxidized. Focused‐ion beam (FIB)‐SEM was used to examine the morphological changes in the cathode composite cross‐sections (**Figure S9**) after cycling. Cracking occurs for both bare and coated CAM particles, but it is much less pronounced in the coated CAM. The fractures due to phase transitions occur mostly at the center of the particle and spread out radially, consistent with previous reports.^[48]^ The volume changes that occur in CAMs are not likely to be sufficiently mediated by a relatively thin coating, as in the case of LiPOF. However, the NCM particle cracking may be mitigated by suppressing side reactions and O_2_ release at the NCM/SE interface during cycling.

Overall, the combination of EIS, ToF‐SIMs, and XPS results unequivocally demonstrate that the LiPOF coating significantly inhibits the oxidative decomposition of the Li_6_PS_5_Cl. Upon cycling, the LiPOF coating further decomposes into stable products such as LiF and LiP_x_O_y_F_z_, forming a stable CEI with the CAM, which underlies the much‐improved performance of the LiPOF‐coated NCM85 cells.

## Conclusions

In this proof‐of‐concept work, by assessing the potential interfacial products between the CAM and the coating material (LiPOF) using DFT, we have rationally designed a stable CEI layerthat can effectively protect against electrochemical oxidation of the argyrodite (Li_6_PS_5_Cl) solid electrolyte. Upon cycling, the LiPOF salt decomposes into LiP_x_O_y_F_z_, LiP_x_F_y,_ and LiF as confirmed experimentally using XPS. These products have a wide electrochemical window and low electronic conductivity, making them ideal interfacial species. Moreover, the simple coating process is conducted through a solution route to produce a nanometric (~30 nm) and conformal coating on the CAM. The treatment procedure is conducted entirely in an ambient atmosphere, obviating the need for sintering at elevated temperatures. This increases the viability of mass production at low cost. The coating effectively suppresses the reaction between the sulfide SE and the NMC85 as confirmed by EIS, XPS, and ToF‐SIMs. As a result, the LiPOF‐coated NMC85 provides a much better rate performance and stability than the uncoated CAM. After 200 cycles at a 0.2 *C* rate, the cell retained 81 % of its initial capacity, while a high‐loading ASSB showed a reversible areal capacity over 4.4 mAh.cm^−2^ and retained 77 % of its initial capacity after 200 cycles at 0.2 *C*.

In summary, our study offers a rational approach for coating design. This work demonstrates the importance of designing stable CEIs based on the decomposition products of the employed coating material, and shows that LiPOF is a suitable material to suppress interfacial degradation in sulfide‐based composite cathodes.

## Conflict of Interests

The authors declare no competing financial interest.

1

## Supporting information

As a service to our authors and readers, this journal provides supporting information supplied by the authors. Such materials are peer reviewed and may be re‐organized for online delivery, but are not copy‐edited or typeset. Technical support issues arising from supporting information (other than missing files) should be addressed to the authors.

Supporting Information

## Data Availability

The data that support the findings of this study are available from the corresponding author upon reasonable request.
