# Supplementary material for: Engineering Stable Decomposition Products on Cathode Surfaces to Enable High Voltage All‐Solid‐State Batteries
Source: Angew Chem Int Ed Engl. 2024 Dec 4;64(2):e202413591. doi: 10.1002/anie.202413591 (PMC11720407; doi:10.1002/anie.202413591)
Supplement: Supplementary file 1 — Supporting Information [file ANIE-64-e202413591-s001.pdf]

Angewandte Chemie
Eine Zeitschrift der Gesellschaft Deutscher Chemiker

Supporting Information

Engineering Stable Decomposition Products on Cathode Surfaces to Enable High Voltage All-Solid-State Batteries

*L. Qian, Y. Huang, C. Dean, I. Kochetkov, B. Singh, L. Nazar**

Supporting Information for:

Engineering Stable Decomposition Products on Sulfide Electrolytes to Enable High Voltage Cathode All-Solid-State Batteries

Lanting Qian[#], Yangyang Huang[#], Cameron Dean, Ivan Kochetkov, Baltej Singh, Linda Nazar*

Department of Chemistry, Waterloo Institute of Nanotechnology, University of Waterloo, Ontario, N2L 3G1, Canada.

# Lanting Qian and Yangyang Huang contributed equally.

Experimental Section

Synthesis

Commercial $LiPO_2F_2$ was purchased from MSE Supplies LLC, and the Li_6PS_5Cl (D50 ~1 μm) powder was purchased from Ampcera Inc. The cathode-active material, $LiNi_{0.85}Mn_{0.1}Co_{0.05}O_2$ (NMC85, D50 ~ 4 μm) was acquired from BASF. The preparation of the $LiPO_2F_2$ coated NMC85 was as follows: first, under argon, the $LiPO_2F_2$ was dissolved in dimethyl carbonate (DMC) and stirred for 2 hours to fully dissolve the material. NMC85 was added to the solution, and the mixture was stirred for 2 hours. The solution was evaporated at 100 °C under controlled nitrogen flow (no additional sintering required). The evaporated sample was finally dried at 100 °C for 6 hours using a BUCHI oven connected to a vacuum line to remove the solvent completely.

Powder X-ray Diffraction

Powder X-ray Diffraction (XRD) measurements on all materials were conducted at room temperature on a PANalytical Empyrean diffractometer equipped with a Pixcel bidimensional detector using Cu–Kα radiation. XRD patterns were obtained in Bragg-Brentano geometry, with samples placed on a zero-background sample holder in an Ar-filled glovebox and protected by Kapton film.

Microscopy Measurements

Surface morphologies of the coated cathode were examined using a Zeiss field emission scanning electron microscope. High-resolution TEM images, EDX mapping, and EELS spectra were collected with a JEM-F200 S/TEM microscope equipped with a single 100 mm^2 SDD detector. FFT transformation, image process, and d-spacing were measured using GMS Digital Micrograph software. FIB-SEM images were collected using a Zeiss Auriga 40 SEM/FIB instrument with a Ga liquid metal ion source. A carbon layer was initially deposited on to the composite cathode to protect the surface prior to FIB cutting. The cross section of the NCM particles was sequentially polished with the FIB beam until a smooth surface was obtained.

X-ray Photoelectron Microscopy and Time-of-Flight Secondary Ion Mass Spectrometry

XPS was performed using a Thermo Fisher K-Alpha XPS instrument with dual turbo molecular pumps and a monochromated and low-power Al K-Alpha X-ray source with a 128-channel detector to enable rapid acquisition. ToF-SIMS spectra were acquired using an ION-TOF ToF-SIMS 5 machine in negative-ion or positive-ion mode with a 30 keV cluster primary ion gun over an area of 200×200 μm^2. 256×256 pixels were used to raster this area. The principal ion current was approximately 0.3 pA. The stop condition was set to 5×10^{12} ions per cm^2 for semiquantitative analysis. The relative intensity of the secondary-ion signals was normalized to the total ion signals, and all signal intensities were collected from the corresponding normalized secondary-ion images.

Electrochemical Measurements

All-solid-state batteries comprised of commercial Li_6PS_5Cl solid-state electrolyte, bare or $LiPO_2F_2$-coated NMC85 cathodes and an Li-In alloy anode were assembled in an argon-filled glovebox. A PEEK cylinder was filled with 120 mg of Li_6PS_5Cl powder and subjected to a pressure of 250 MPa for one minute. The composite cathode formulations were fabricated by grinding Li_6PS_5Cl with bare or $LiPO_2F_2$ coated NCM85 in an agate mortar at a weight ratio 2:8 for ~ 15 minutes. Composite cathodes were spread and compressed on top of the Li_6PS_5Cl at a pressure of 200 MPa for ~3 minutes. A thin indium foil with a diameter of 10 mm and a thickness of 0.1 mm was affixed to the opposite side of the Li_6PS_5Cl pellet. Approximately 1.5 mg Li, which had been flattened over copper foil, was positioned over the indium foil to form the LiIn alloy as the anode. A load cell was utilized to determine the initial applied pressure of 200 MPa before insertion of the cell into the stainless-steel casing. The cells underwent galvanostatic cycling using a VMP3 (Bio-Logic) or MACCOR cycler within the voltage range and rate specified in the figures (NCM85, 1C = 200 $mA \cdot g^{-1}$). The EIS was quantified at a temperature of 25°C utilizing a Bio-

Logic SP-200 potentiostat across a frequency spectrum of 100 mHz to 1 MHz. RelaxIS software was utilized to perform EIS fitting of the results.

Calculation of Areal Capacity:

For coated LiPOF-NCM cell at a loading of 9.9 $mg.cm^{-2}$ (active material)

Areal capacity = 182 $mAh.g^{-1} \times 0.0099$ $g.cm^{-2}$ = 1.8 $mAh.cm^{-2}$

For coated LiPOF-NCM cell at a loading of 25.6 $mg.cm^{-2}$ (active material)

Areal capacity = 173 $mAh.g^{-1} \times 0.0256$ $g.cm^{-2}$ = 4.4 $mAh.cm^{-2}$

X-ray Absorption Spectroscopy

XANES spectra were collected at the Spherical Grating Monochromator (SGM) beamline at the Canadian Light Source (CLS) using a four-silicon-drift-detector array. The samples were transferred through a special sealed chamber from the glovebox to ensure an air-free environment. Background subtraction and normalization were conducted using Athena.

Computational Methods

Density functional theory (DFT) calculations were used to examine the $LiPO_2F_2$ coating. All calculations used VASP input parameters that matched those used by Materials Project for energy minimization: an energy cutoff of 520 eV, Monkhorst-Pack k-point mesh of 1000 per reciprocal atom, and energy convergence criterion of 0.0005*(number of atoms) eV per cell. Using these parameters and the crystal structure of $LiPO_2F_2$, we calculated the 0 K DFT energy. To evaluate the thermodynamic stability of $LiPO_2F_2$, DFT energies for other materials in the Li-P-O-F system were extracted from the Materials Project database. This dataset was then supplemented with additional structures from the Inorganic Crystal Structure Database, where the DFT energies of these structures were determined similarly to the calculation for $LiPO_2F_2$. With this dataset compiled, the Python Materials Genomics (Pymatgen) package was used to generate the element profile of $LiPO_2F_2$ with respect to the applied potential.

Table S1. Summary of voltage window and bandgaps of the interfacial species obtained from DFT.

Composition	Voltage Window (vs Li^+/Li)	Band Gap (eV)
LiF	**0-6.3 V (1)**	**8.7**
LiCl	**0-4.3 V (1)**	**6.3**
$LiPO_3$	**2.5-4.9 V (2)**	**2.5**
Li_3PO_4	**0-5.0 V (3)**	**5.8**
$Li_4P_2O_7$	**2.3-4.3 V**	**5.6**
Li_2NiF_4	**2.8-5.6 V**	**4.7**
P_2S_5	**2.3 V-**	**2.6**
$P_2O_3F_4$	**3.1 V-**	**5.8**
$SOCl_2$	**3.3 V-**	**3.6**
P_2S_7	**2.3 V-**	**2.1**
CoO	**1.9 V-**	**0.6**
CoO_2	**4.1 V-**	**0.6**
MnO_2	**3.7 V-**	**0.5**
$Ni_3(PO_4)_2$	**2.8 V-**	**3.4**

*Note that P_2S_5 and subsequent entries do not contain Li and thus have no upper voltage limit

List of Supporting Figures

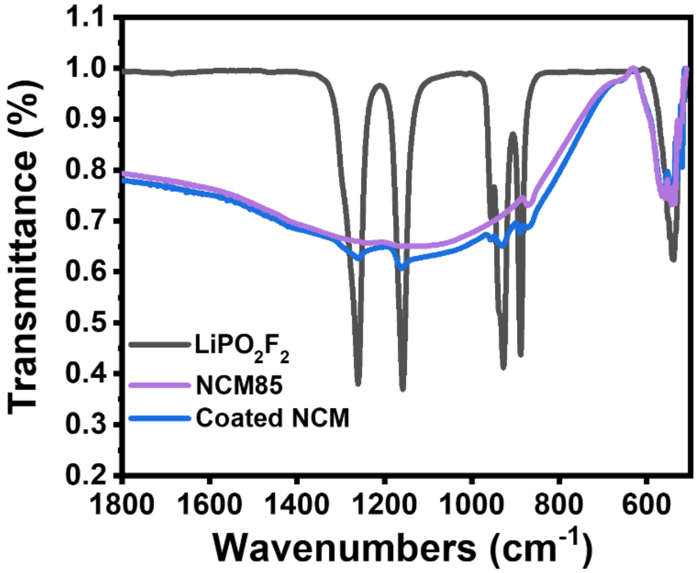

Figure S1. FTIR spectra of bare NCM85, $LiPO_2F_2$, and $LiPO_2F_2$-coated NCM85.

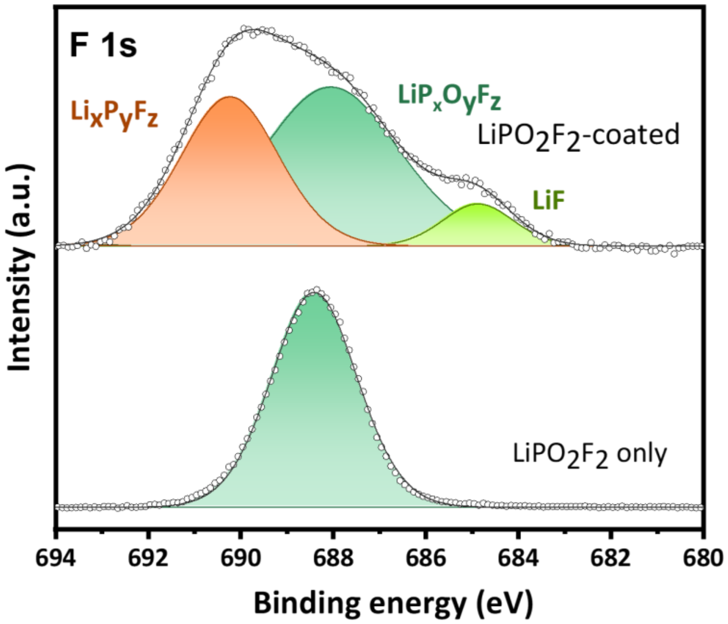

Figure S2. F 1s XPS spectra of $LiPO_2F_2$-coated NCM85 and $LiPO_2F_2$.

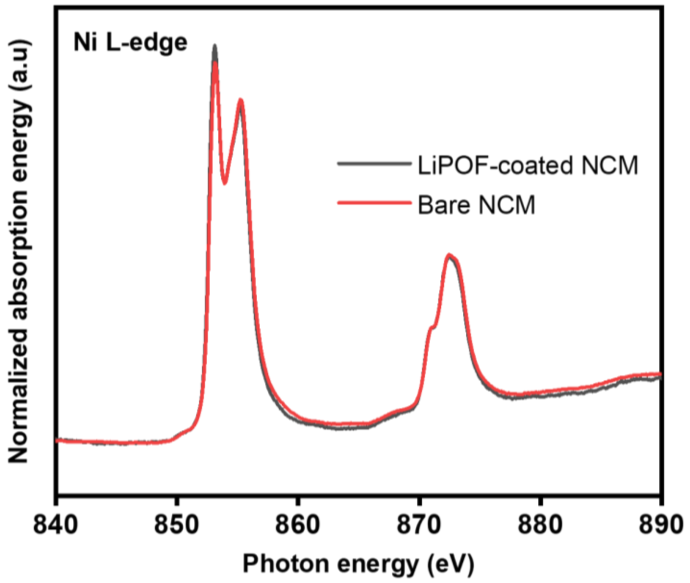

Figure S3. XANES Ni L-edge of the coated and uncoated CAM before cycling.

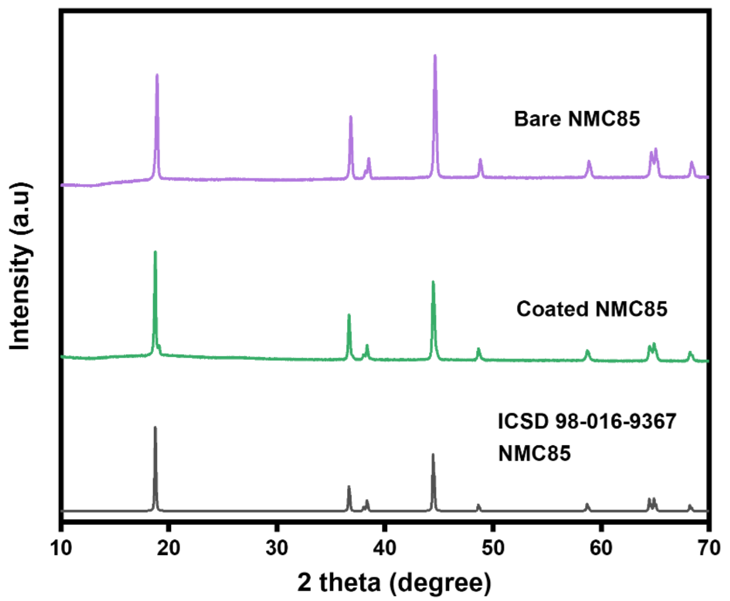

Figure S4. XRD patterns of bare NCM85 and LiPOF-coated NCM85.

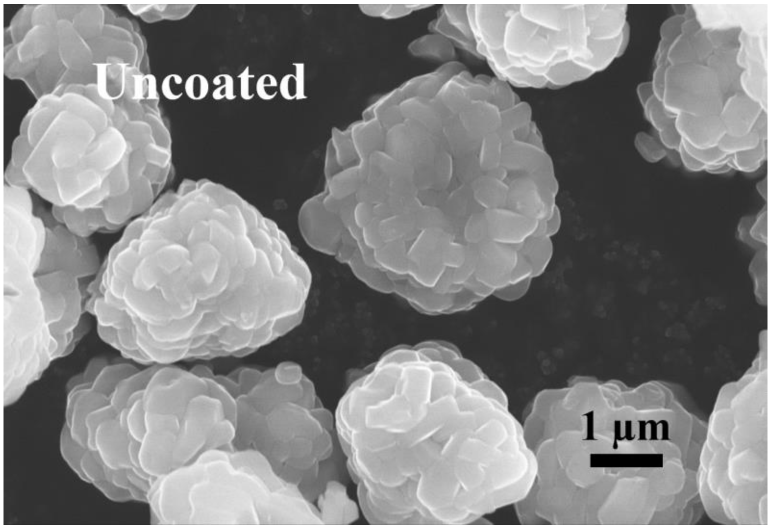

Figure S5. SEM image of the uncoated NCM85 particles.

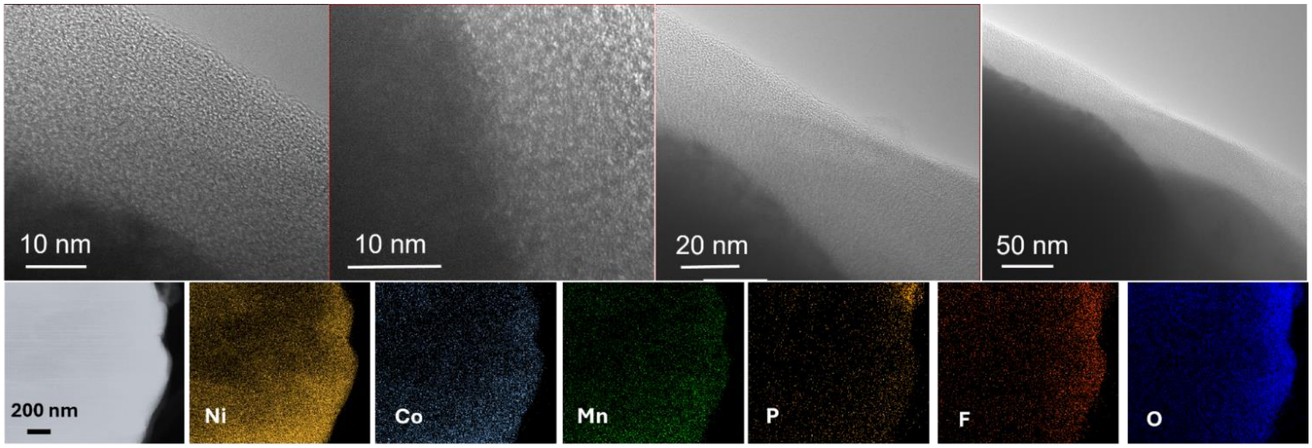

Figure S6. Additional HR-TEM images and STEM-EDX of LiPOF-coated NCM85 particles.

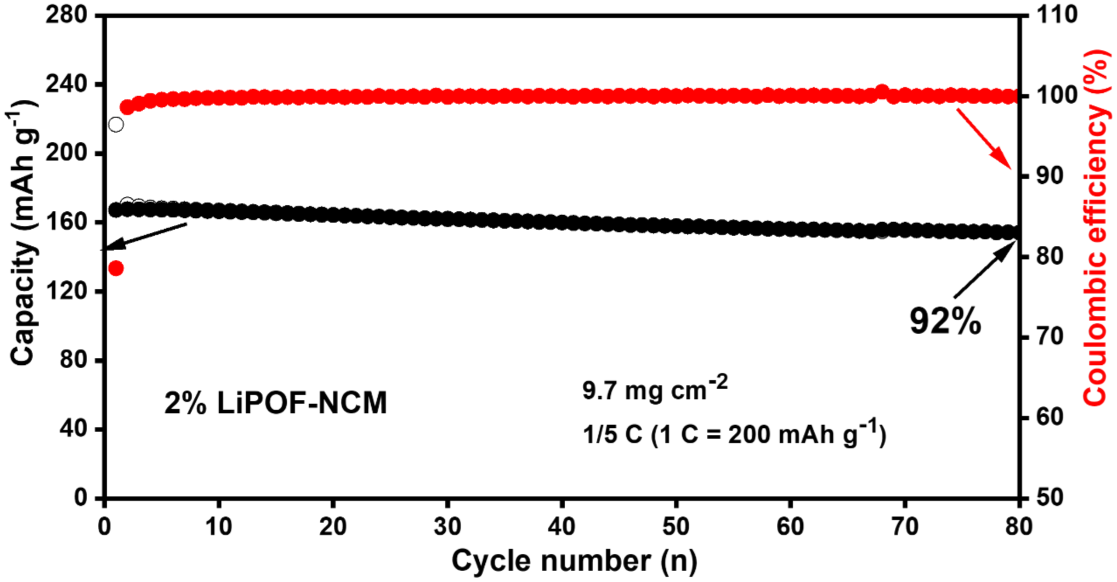

Figure S7. Electrochemical performance of 2 wt% LiPOF-coated NCM cell cycled at room temperature.

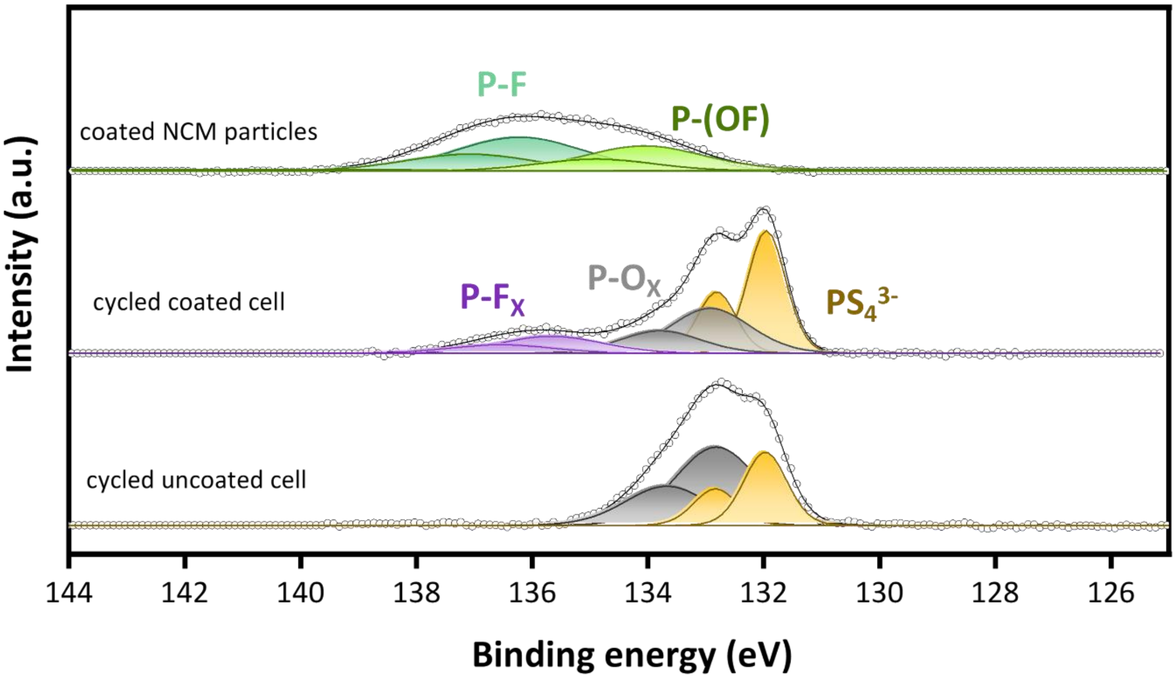

Figure S8. P *2p* XPS of uncycled LiPOF-coated NCM particles, cycled composite cathode for both uncoated and coated cells (after 200 cycles at 0.2 *C*). These data show that P *2p* XPS spectra cannot definitively identify the formation of $LiPO_3$ species, because the coating and any partially oxidized argyrodite that might be formed (if the coating wasn't ideal) both contain P-O_x species. The P $2p_{3/2}$ signal from $LiPO_3$ would appear at a binding energy of 133 eV (the same as reported for Li_3PO_4; NIST database). These are usually deemed "PO_x" in the literature and are also where P-O_x signals from oxidized Li_6PS_5Cl would appear. However, the almost complete lack of oxidized sulfide in the S 2p spectrum (Fig 4b, main text), indicates that the argyrodite is *not* oxidized, and hence the P-O_x species in the cycled coated cell likely arises from $LiPO_3$.

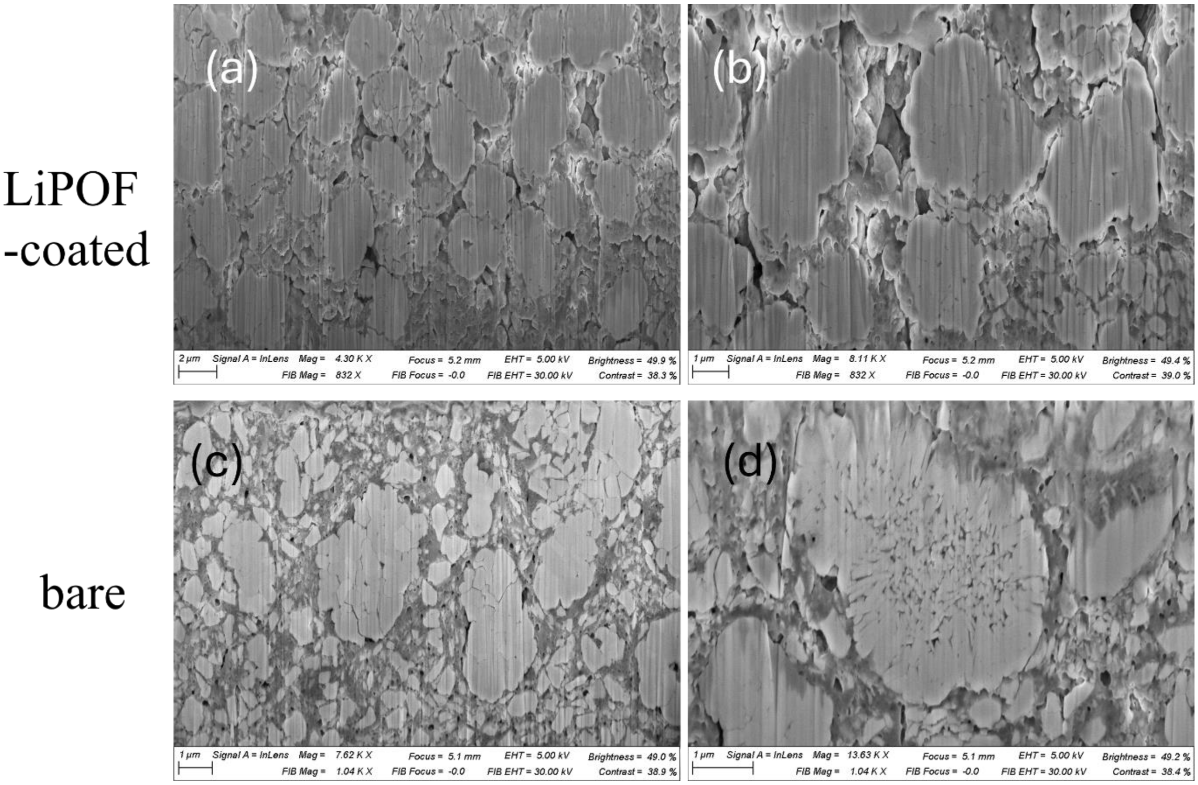

Figure S9. FIB cross-section images of bare and LiPOF-coated cells after 200 cycles at 0.2*C*. (a, b) Low and high-resolution SEM images of the LiPOF-coated NCM cathode; (c, d) Low and high-resolution SEM images of the bare NCM cathode.
